# Genomics and Drug Discovery Strategies: The Role of Natural Compounds and Its Receptor in Alzheimer’s Disease

**DOI:** 10.7759/cureus.52423

**Published:** 2024-01-17

**Authors:** Shweta Mishra, Tarique Aziz, Annie J Toppo, Daksh Kumar, Mona P Tirkey, Priyangulta Beck, Nawed Anjum, Dipanjali Sharma, Md. Mahfooz Khan, Shristi Kumari, Pinki R Sahu, Mukesh Nitin

**Affiliations:** 1 Genetics, Digianalix, Ranchi, IND; 2 Biotechnology, Marwari College, Ranchi, IND; 3 Biochemistry, Rajendra Institute of Medical Sciences, Ranchi, IND

**Keywords:** computational approach, neurodegenerative diseases, md simulation, next generation sequencing, docking

## Abstract

Alzheimer’s Disease (AD) is a special class of neurodegenerative diseases demarcated as a progressive disorder affecting especially older adults globally. The AD-infected brain shows declination in cognitive functions, memory loss, and other exhausting symptoms. In this study, we focused on using advanced bioinformatics and next-generation sequencing to explore essential clusters of genes from various diversified Alzheimer’s, Parkinson and Frontotemporal Dementia diseased cases. The significant differential expression analysis of genes (p-value ≤ 0.05, log fold change ≤ 0.05) was carried out, followed by meta-analysis, which resulted in the identification of 20 conserved genes across variable case studies. Out of 20 conserved genes, CASP8 and PTPN11 were observed to show essential regulatory mechanisms in AD metabolic pathways and proceeded further for docking analysis.

Moreover, the natural compounds were screened for ligand library preparation based on extensive scientific literature and (ADMET (Absorption, Distribution, Metabolism, Excretion, and Toxicity)) property check. Molecular docking was carried out with screened ligands and target receptors, resulting in the identification of Rosmarinic acid (RA) with CASP8 having docked score (∆G = -8.0 kcal/mol); Donepezil (FDA drug) dock score (∆G = -7.3 kcal/mol) (control). PTPN11 receptor with Carnosol ligand resulted in docking score (∆G = -9.1 kcal/mol) w.r.t Tacrine (FDA drug) docked score (∆G = -8.0 kcal/mol) followed by MD simulation. This research will aid in the identification of potential natural compounds that future researchers can use for further validation as a potential candidate drug in combating various neurodegenerative diseases highlighting AD.

## Introduction

In recent trends, the lifestyles of people are so stressful that they disturb the cognitive functions of the brain, leading to AD. AD is a neurodegenerative inflammatory disorder leading to degeneration of the brain cells in two classified ways: (a) formation of the β-amyloid (Aβ) plaques in the neurons around the nucleus in the medial temporal lobe of the brain and (b) formation of the tau (τ) tangles in the neurofibrillary region causing the loss of many neurons from different part of the brain cells [1]. AD is a progressive disease where multiple factors may be responsible, such as mental illness, genetic factors, injury to the head, infections, environmental factors, and such. Except for dementia, other multiple indicators show a low supply of oxygen in the brain, dietary deficiency, and the occurrence of tumors in certain parts, which leads to the decline of brain activeness to nothing. Generally, it is diagnosed in people aged above 65 years and is categorized as Late Onset AD (LOAD), but it is also diagnosed in some people with age less than 65 years and termed Early Onset AD. (EOAD) [2]. Several research studies have been done and are still proceeding to find a suitable or worthy cure for the disease.

Neuroinflammation is one of the major symptoms and needs the involvement of glial cells like astrocytes, microglial cells, and oligodendrocytes to preserve the central nervous system (CNS). As stated before, due to the formation of Aβ plaques in the medial temporal lobes and tau tangles in the neurofibrillary region of the neuron, the microglial cells (forms 10% of the CNS cells) from deactivated or dormant phase to activated phase wherein the process of phagocytosis acts upon all the Aβ and tau tangles [3] and these microglial cells secretes numerous proinflammatory such as IL-1β, IFN, ROS, RNS, COX-1, COX-2, TNF-α, IL-6, and many more cytokines and chemokines [4].

Certain neuroinflammatory features are assigned in the triggering of cells like microglia, astrocytes, and other acquired immunity located in the brain parenchyma, and the appearance of some inflammatory intermediaries like cytokines, chemokines, and so on [5]. The current introduction of advanced computational and next-generation sequencing has resulted in the identification of various important genes involved in the contribution of metabolic pathways to neurodegenerative diseases [6]. Further, using the genomic-based approach, we tried to identify a group of hub genes obtained from various cases of neurodegenerative diseases. The PTPN11 gene is important as it shows various gene expressions in different cases of AD. It also plays a multi-functional role in the cell cycle and controls the functioning of nerve growth factors, leading to neurogenesis [7]. While the CASP8 gene is identified as a key player in cellular differentiation, apoptosis, and inflammation functions. Mediation of Aβ-induced neuronal apoptosis, activation of microglial cells, and cleaving of a protein called BID, leading to the release of cytochrome-c and causing mitochondrial damage and TNF signaling pathways, are all regulated by the CASP8 gene [8]. Moreover, using structural bioinformatics and a drug design approach, we identified potential target receptors.

Despite the fact that there is no established cure for AD, however, the effects and progressions of the disease can be controlled to a limited extent for a while with the usage of certain natural compounds present in the market approved by the Food and Drug Administration (FDA). Similarly, other metabolites reported in various literature can be considered potential drug compounds targeting various neurodegenerative diseases. In our study, we tried to explore vital gene expressional behavior for variable neurodegenerative diseases using metagenomics analysis. Moreover, we identified potential target receptors from vital hub genes and were further screened against various naturally selected potential compounds in order to explore a candidate drug, resulting in futuristic research highlighting the possibility of curing AD.

## Materials and methods

Sample collection and computational approach

During our studies, the datasets were collected from an open-source genomic database (GEO Dataset, NCBI) targeting various neurodegenerative diseases like Alzheimer's Disease (AD), Frontotemporal Dementia (FTD), and Parkinson's Disease (PD). In our study, we considered three different genomic data cases of variable AD, FTD, and PD represented as Case 1 (GSE138260) represents expression analysis of the data from infected brains from AD patients and normal brains from healthy humans in 36 different samples, Case 2 (GSE13162) shows the expression of brain collected from postmortem with or without FTD infection in 56 samples and Case 3 (GSE19587) represents the presence of some certain molecular markers that are present in the brain of patients with PD or not with a total number of 22 samples. The datasets were re-analyzed using the GEO analyzer NCBI server [9]. Further, the analysis resulted in the generation of a box plot, volcano plot, and MA plot along with a gene expression table using the Benjamin-Hedge Hoch test and limma package-R software. The expression genes' filtration was carried out with the stringent cut-off score of P-value < 0.05 and the log fold chain (Log FC) < 0.05 using an in-house shell script for all the 3 different cases.

System biology approach and identification of hub genes

The conservancy analysis of the genes across all three different neurodegenerative diseases was analyzed using the Venn Ghent server [10]. The screened genes were further imported into Cytoscape [11] for the protein-protein interaction network studies. The network was re-analyzed using the Cytoscape package to identify the top 20 hub genes.

Homology modeling and Receptor preparation

Two of the top 20 hub genes, CASP8 and PTPN11, were identified as vital genes responsible for various metabolic pathways related to AD [7,8]. The gene sequences were downloaded from UniProt and were imported to the Swiss Model server [12] for homology modeling prediction. Then, physiochemical properties evaluation for the predicted proteins was carried out using ProtParam [13], Ramachandran plot using PROCHECK [14], secondary structure prediction using Self-Optimized Prediction method with Alignment (SOPMA) [15], and transmembrane structure prediction using SOSUI server [16]. The final structure proteins of CASP8 and PTPN11 were used for receptor target prediction with CHIMERA-Auto dock Vina [17] by removing ions, ligands, and solvents previously present in the structures. Also, the addition of H-bond and energy minimization of receptor proteins was accomplished by Swiss PDB Viewer (SPDBV) [18], resulting in final target receptor preparation.

Screening of compounds and ligand preparation

The important natural metabolites library preparation was based on various bibliographic literature and was downloaded from the PubChem database. The collected metabolites were screened for potential ligand compounds based on ADMET properties using DruLiTo [19] having parameters like MV, logP, AlogP, HBA, HBD, TPSA, AMR, nRB, nAtom, nAcidic Group, RC, nRigidB, nArom Ring, nHB, SAlerts and Osiris [20] qualifying properties like tumorigenic, irritant, mutagenic or reproductive effective. The final ligands passing OSIRIS and DruLiTo were used in Avogadro [21] and Chimera for ligand-receptor drug docking studies.

Molecular docking and MD simulation

The docking analysis was carried out with CASP8 and PTPN11 receptors and potential ligands using CHIMERA-Auto dock Vina. As an outcome of docking, it was revealed that the receptors and ligands showed minimum binding energy (-ΔG), leading to the prediction of probable drug ligands. Further, the docking score was critically analyzed and visualized using Biovia Discovery Studio [22], followed by molecular dynamics simulation (MD) with iMOD [23].

## Results

Computational-based genomic analysis of conserved genes targeting neurodegenerative disorders

**Figure 1:**
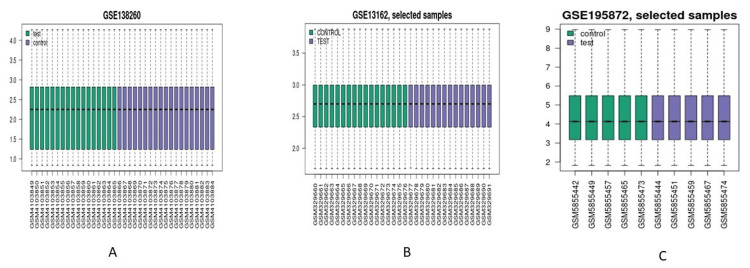
Normalization plot from three different cases based on the meta-analysis done for Geo Datasets ID number (A) GSE138260 (B) GSE13162 (C) GSE195872 w.r.t control vs test samples.

**Figure 2:**
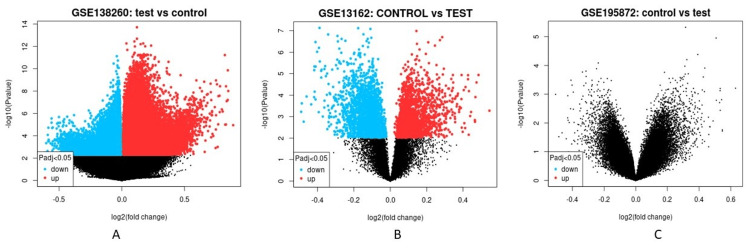
Volcano plot from three different cases based on the meta-analysis done for Geo Datasets ID number (A) GSE138260 (B) GSE13162 (C) GSE195872 w.r.t control vs test samples.

**Figure 3:**
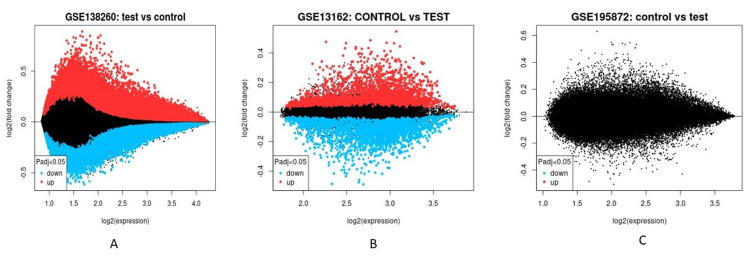
MA plot from three different cases based on the meta-analysis done for Geo Datasets ID number (A) GSE138260 (B) GSE13162 (C) GSE195872 w.r.t control vs test samples.

In order to explore the gene activities in AD, FTD, and PD, several cases of the neurodegenerative disease were screened, and a total of 3 variable cases were selected for metagenomic analysis. All the raw reads of selected samples were downloaded and re-analyzed for read normalization demarcated as tests and controls represented in the box plot, volcano plot, and MA plot (Figures 1-3).

**Figure 4:**
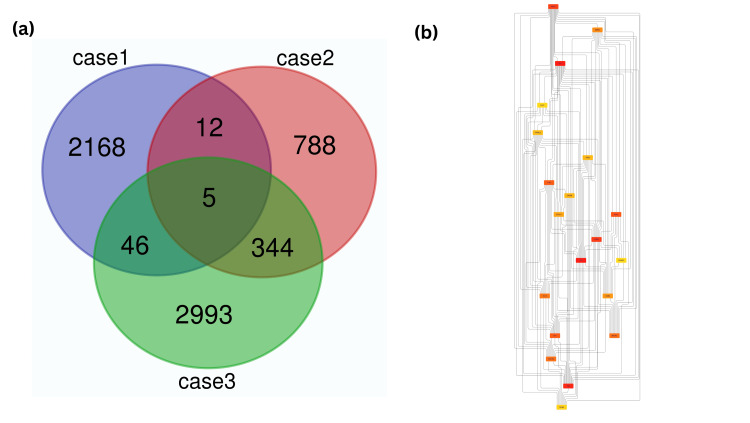
(a) Venn diagram for the three different cases showing conserved genes in the overlapping regions (b) String analysis graph with PPI network of top twenty expressed genes from the three cases.

**Figure 5:**
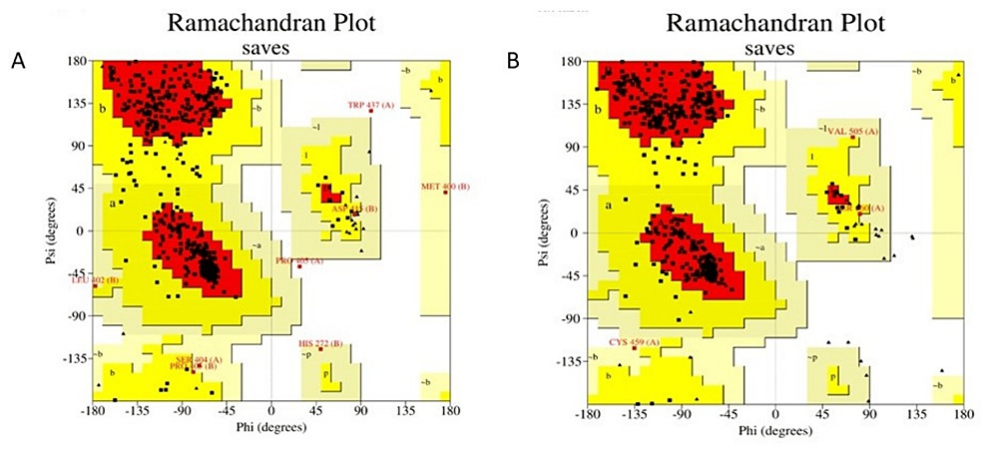
Ramachandran plot specifying the presence of amino acid residues in most favorable, additional allowed, generously allowed and disallowed regions in proteins for (A) CASP8 (B) PTPN11.

Further significant differentially expressed genes (DEGs) (p-value <0.05 and logFC <0.05) analysis was carried out, resulting in the identification of a total of 2231, 1149, and 3388 genes from case 1 (GSE138260), case 2 (GSE13162), and case 3 (GSE19587) respectively. The screened significant genes were subjected to conservancy analysis across three different neurodegenerative cases (AD, FTD, and PD). A total of 407 genes, like SUMO1, SRC, BRCA1, TNF, etc., were revealed as an outcome among the three conserved cases, as represented in (Figure 4a). The assessment of conserved genes was further visualized in open-source software to observe the protein-protein interactions network influencing metabolic pathways w.r.t our diseases. Among the top occurring genes TP53, ITGB3, KRAS, SYK, CASP8, PTK2, PTPN11, etc. (Figure 4b), two of them, CASP8 and PTPN11, have well-defined metabolic pathways highlighting the reduction of progression in AD, hence their homology modeling was carried out resulting in GMQE value 0.37 for CASP8 whereas 0.90 for PTPN11. Ramachandran plot for CASP8 and PTPN11 is shown in (Table 1) and (Figure 5).

**Table 1:** Depiction of residues in the different regions of the receptors CASP8 and PTPN11.

RAMACHANDRAN PLOT ANALYSIS	CASP8	PTPN11
Residue in most favourable regions (A, B, L)	86%	90.7%
Residues in additional allowed regions (a, b, l, p)	12.6%	8.7%
Residues in generously allowed regions (~a, ~b, ~l, ~p)	1.1%	0.6%
Residues in disallowed regions	0.2%	0.0%

**Figure 6:**
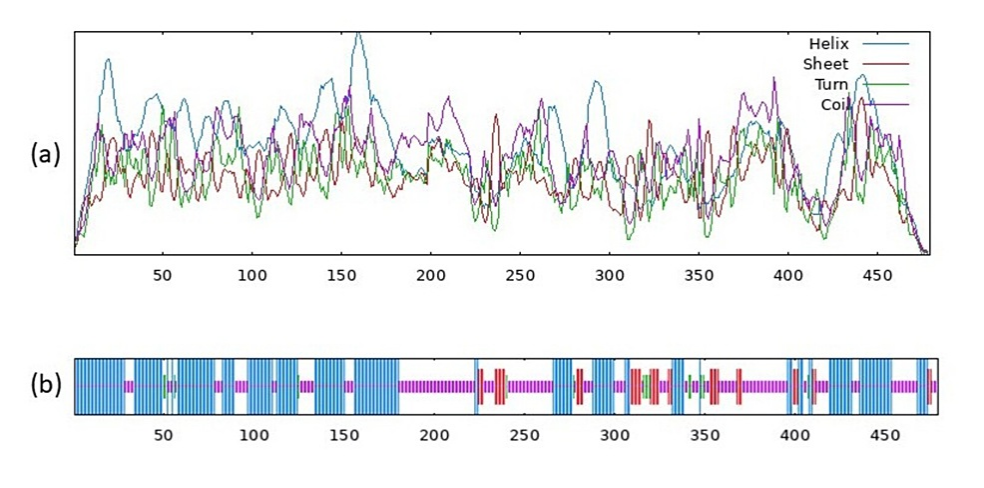
Structure of Caspase-8 protein. (a) Visual representation of the predicted states (b) Secondary structure prediction of the Caspase-8 with score curves for predicted states.

**Figure 7:**
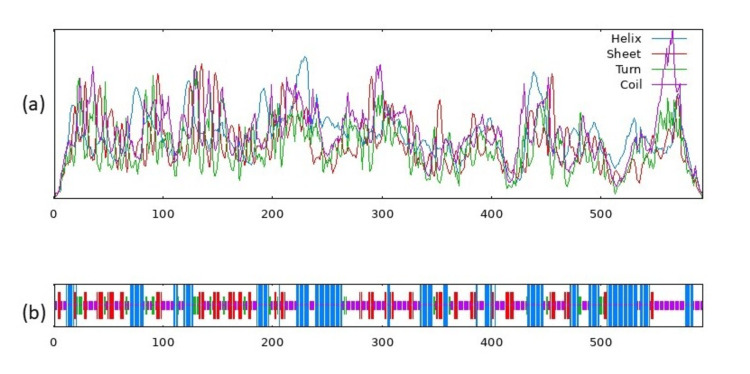
Structure of the Tyrosine-protein phosphatase non-receptor type 11 protein. (a) Visual representation of the predicted states (b) Secondary structure prediction of the protein with score curves for predicted states.

Physiochemical structural analysis of CASP8 and PTPN11 showed instability index 44.13, 43.07, and hydropathicity (GRAVY) -0.529 and -0.735, respectively, as shown in (Figure 6-7).

**Figure 8:**
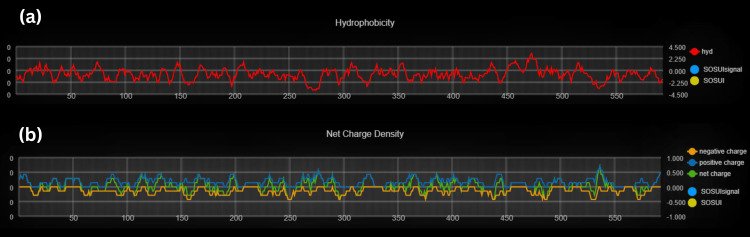
(a) Hydropathy plot indicating hydrophobicity through red line and Sosui region in yellowish part (b) Charge plot of Tyrosine-protein phosphatase non-receptor type 11 protein showing types of charges present such as negative charge through orange line, positive charge through indigo line and net charge through green line and the highlighted yellowish region is Sosui region.

**Figure 9:**
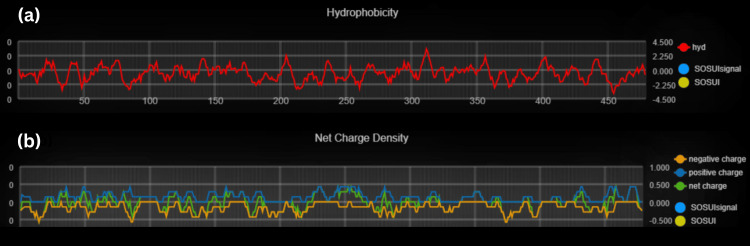
(a) Hydropathy plot indicating hydrophobicity through red line and Sosui region in yellowish part (b) Charge plot of Caspase-8 protein showing types of charges present such as negative charge through orange line, positive charge through indigo line and net charge through green line and the highlighted yellowish region is Sosui region.

Secondary structural and transmembrane prediction resulted in the identification of CASP8, the sequence length of 479 amino acids (aa), and a similarity threshold of 8, whereas PTPN11 has a 593 aa sequence length, similarity threshold of 8 as represented in (Table 2) and (Figure 8-9).

**Table 2:** Prediction of the nature of the target protein via SOSUI.

S No.	Protein	Region	Transmembrane seq.	Type
1	CASP8	396-418	CCCCCCCCCCCCCCCCCCCCCCC	Primary
2		482-503	TTFEELHFECCCCCCCCCCCCC	Secondary
3		523-544	CCCCCCCCCCCCCCCCCCCCCC	Primary
4		793-814	CCCCCCCCCCCCCCCCCCCCCC	Primary
5	PTPN11	1108-1129	EECCCCCCCCCCCPPCTPTPPC	Secondary
6		1148-1169	QQKSFRCCCCCCCCCCCCCCCC	Primary

Target receptor and ligand library construction

The candidate genes CASP8 and PTPN11 were scrutinized for receptor construction by removing additional chains, ions, and solvents. The energy minimization of the receptor was established by adding hydrogen and charge in the gastieger force field for final target receptor preparation. Values of Pocket Id. for CASP8 were X= 5.123, Y= -83.853, and Z= 11.025, while that for PTPN11 was X= 37.886, Y= 23.401, and Z= 23.214 with sizes of 20 each. For docking these receptors, we used ligand library preparation using various plants with herbal and medicinal properties identified based on various scientific articles on AD. The selected herbal plants include- Emblica officinalis, Withania somniferous, Rosmarinus officinalis, Camellia sinensis, Curcuma, Coriandrum sativum, Panax ginseng, Crocus sativus, and Bacopa monnieri. Out of which, a total of 26 metabolites like RA, Carnosol, Carnosic acid etc. were screened for ADMET properties like mutagenic, tumorigenic, and irritant, out of which 16 compounds showed no sign of toxicity as shown in (supplementary Table 5-6).

Docking and MD simulation

**Figure 10:**
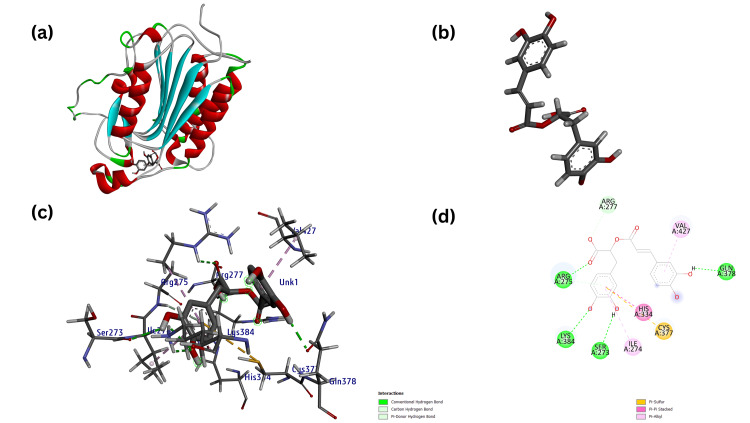
(a) Modeled three-dimensional structure of the receptor CASP8 with ligand Rosmarinic acid (b) Ligand Rosmarinic acid (acting as test) prepared from metabolite through Chimera (c) Receptor-ligand interaction between the CASP8 and Rosmarinic acid and (d) Modeled two-dimensional structure of the CASP8- Rosmarinic acid interaction.

**Figure 11:**
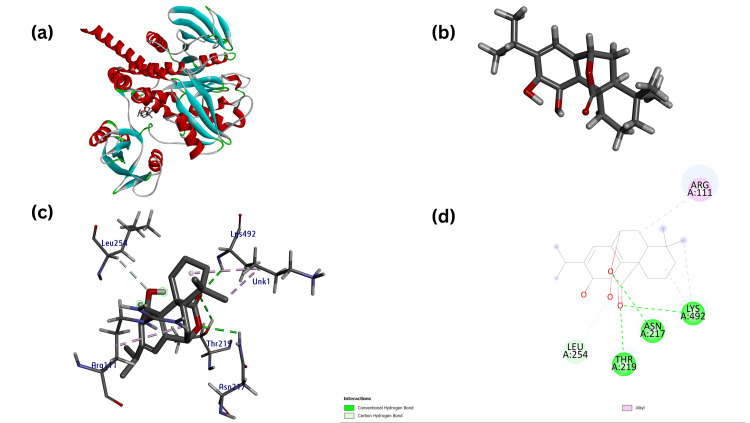
.(a) Modeled three-dimensional structure of the receptor PTPN11 with ligand Carnosol (b) Ligand Carnosol (acting as test) prepared from metabolite through Chimera (c) Receptor-ligand interaction between the PTPN11 and Carnosol and (d) Modeled two-dimensional structure of the PTPN11-Carnosol interaction.

Using final target receptors CASP8 and PTPN11, docking studies were carried out against the final screened ligands, resulting in the identification of the promising drug compound Rosmarinic acid (RA) against CASP8 with a docking score of -8.0 kcal/mol (Figure 10) and that of Carnosol against PTPN11 with a docking score of -9.1 kcal/mol (Figure 11).

**Figure 12:**
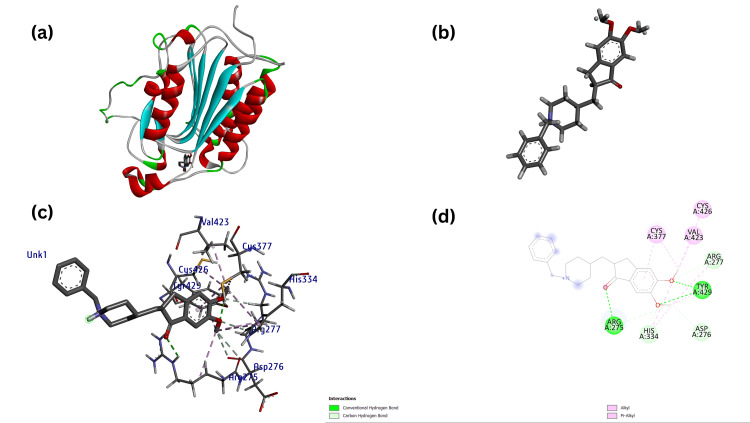
(a) Modeled three-dimensional structure of the receptor CASP8 with ligand Donepezil (b) Ligand Donepezil (acting as control) prepared from metabolite through Chimera (c) Receptor-ligand interaction between the CASP8 and Donepezil and (d) Modeled two-dimensional structure of the CASP8-Donepezil interaction.

**Figure 13:**
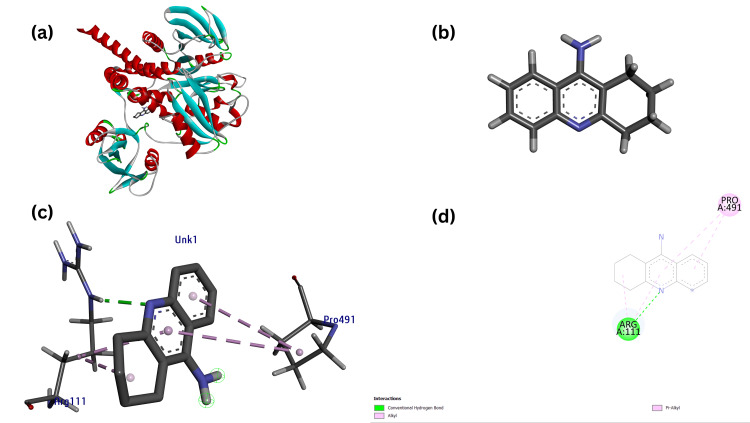
(a) Modeled three-dimensional structure of the receptor PTPN11 with ligand Tacrine (b) Ligand Tacrine (acting as test) prepared from metabolite through Chimera (c) Receptor-ligand interaction between the PTPN11-Tacrine and (d) Modeled two-dimensional structure of the PTPN11-Tacrine interaction.

Similarly, docking scores of CASP8 and PTPN11 were carried out against standard compounds (FDA approved), showing Donepezil against CASP8 with a docking score of -7.3 kcal/mol (Figure 12) and Tacrine against PTPN11 with a docked score of -8.0 kcal/mol (Figure 13) as shown in (Table 3).

**Table 3:** Best docking score/ binding affinity of the ligands (both test and control) against CASP8 and PTPN11 receptors.

S. No.	Receptor	Ligands	Vina score/ Binding affinity (kcal/mol)	RMSD i.b	RMSD u.b
1.	CASP8	Rosmarinic acid (Test)	-8.0	0.0	0.0
2.	CASP8	Donepezil (Control)	-7.3	0.0	0.0
3.	PTPN11	Carnosol (Test)	-9.1	0.0	0.0
4.	PTPN11	Tacrine (Control)	-8.0	0.0	0.0

The RA against the CASP8 receptor resulted in eight H-bonds and four hydrophobic bonds, whereas Carnosol against the PTPN11 receptor resulted in four H-bonds and hydrophobic bonds, as shown in (Table 4).

**Table 4:** Analysis via Molecular Docking: Binding affinity and molecular interaction of the Donepezil (control) and natural metabolite Rosmarinic acid against receptor CASP8; binding energy and molecular interaction of Tacrine (control) and bioactive compound Carnosol against receptor PTPN11.

Receptor-Ligand Interaction	Vina score/Binding affinity (kcal/mol)	Ligands	Distance (A^0^)	Receptor	Category	Types of Interaction
CASP8- Donepezil (Control)	-7.3	UNL1:C	4.38	A:CYS377	Hydrophobic	Alkyl Interaction
		UNL1	5.01	A:CYS377	Hydrophobic	Pi-Alkyl Interaction
		UNL1:C	3.75	A:VAL423	Hydrophobic	Alkyl Interaction
		UNL1:C	4.41	A:HIS334	Hydrophobic	Pi-Alkyl Interaction
		UNL1:C	4.48	A:CYS426	Hydrophobic	Alkyl Interaction
		UNL1:C	4.83	A:HIS334	Hydrophobic	Pi-Alkyl Interaction
		UNL1:C	4.92	A:TYR429	Hydrophobic	Pi-Alkyl Interaction
		UNL1:C	4.55	A:ARG275	Hydrophobic	Alkyl Interaction
		UNL1:C	3.27	A:ARG275:O	H-Bond	Carbon H-Bond Interaction
		UNL1:O	2.53	A:HIS334:HE1	H-Bond	Carbon H-Bond Interaction
		UNL1:C	3.38	A:ASP276:O	H-Bond	Carbon H-Bond Interaction
		UNL1:O	2.64	A:ARG275:HE	H-Bond	Conventional H-Bond Interaction
		UNL1:O	2.30	A:TYR429:H	H-Bond	Conventional H-Bond Interaction
		UNL1:O	2.05	A:TYR429:HH	H-Bond	Conventional H-Bond Interaction
CASP8-Rosmarinic ACID (Test)	-8.0	UNL1:H	2.05	A:ASN381:OD1	H-Bond	Conventional H-Bond Interaction
		UNL1:O	2.72	A:GLN378:HE22	H-Bond	Conventional H-Bond Interaction
		UNL1:O	2.26	A:LYS384:HZ2	H-Bond	Conventional H-Bond Interaction
		UNL1:O	2.51	A:LYS384HZ1	H-Bond	Conventional H-Bond Interaction
		UNL1	4.94	A:CYS377:SG	Hydrophobic	Pi-Sulphur Interaction
		UNL1	5.09	A:ARG275	Hydrophobic	Pi-Alkyl Interaction
		UNL1	5.04	A:ILE274	Hydrophobic	Pi-Alkyl Interaction
		UNL1	5.07	A:HIS334	Hydrophobic	Pi-Pi Stack Interaction
		UNL1:O	2.76	A:ARG275:HH12	H-Bond	Conventional H-Bond Interaction
		UNL1:O	3.04	A:ARG275:HE	H-Bond	Conventional H-Bond Interaction
		UNL1:O	2.72	A:TYR429:HH	H-Bond	Conventional H-Bond Interaction
		UNL1:O	2.96	A:ARG277:HH12	H-Bond	Conventional H-Bond Interaction
PTPN11-Tacrine (Control)	-8.0	UNL1	5.37	A:PRO491	Hydrophobic	Pi-Alkyl Interaction
		UNL1	5.49	A:PRO491	Hydrophobic	Pi-Alkyl Interaction
		UNL1:N	2.22	A:ARG111:HE	H-Bond	Conventional H-Bond Interaction
		UNL1	4.12	A:ARG111	Hydrophobic	Pi-Alkyl Interaction
		UNL1	3.88	A:ARG111	Hydrophobic	Alkyl Interaction
PTPN11-Carnosol (Test)	-9.1	UNL1:O	2.99	A:LYS492:HN	H-Bond	Conventional H-Bond Interaction
		UNL1:O	2.60	A:THR219:HG1	H-Bond	Conventional H-Bond Interaction
		UNL1	4.54	A:LYS492	Hydrophobic	Alkyl Interaction
		UNL1:C	3.83	A:LYS492	Hydrophobic	Alkyl Interaction
		UNL1:O	2.68	A:ASN217:HD22	H-Bond	Conventional H-Bond Interaction
		UNL1:O	3.09	A:LEU254:HA	H-Bond	Conventional H-Bond Interaction
		UNL1	4.57	A:ARG111	Hydrophobic	Alkyl Interaction

**Figure 14:**
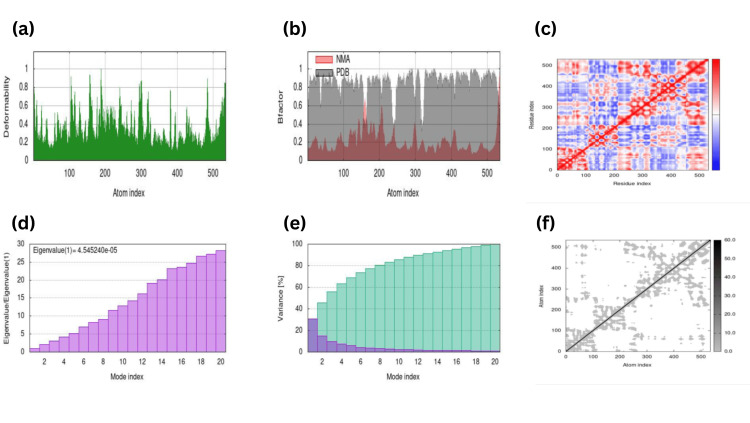
Molecular dynamic simulation done through iMODS of the Rosmarinic acid against CASP8 receptor depicting (a) Deformability showing 10 hinges, 5 of them between 0.8 to 1, 4 hinges between 0.6 to 0.8 and 1 hinges between 0.4 to 0.6 (b) Bfactor showing almost overlapping between predicted and experimental graph (c) Eigenvalue(1)=4.54e-05 (d) Variance (e) Covariance showing correlated regions around the central line in red (f) Elastic network in grey around the central line more flexibility meaning less deformability.

**Figure 15:**
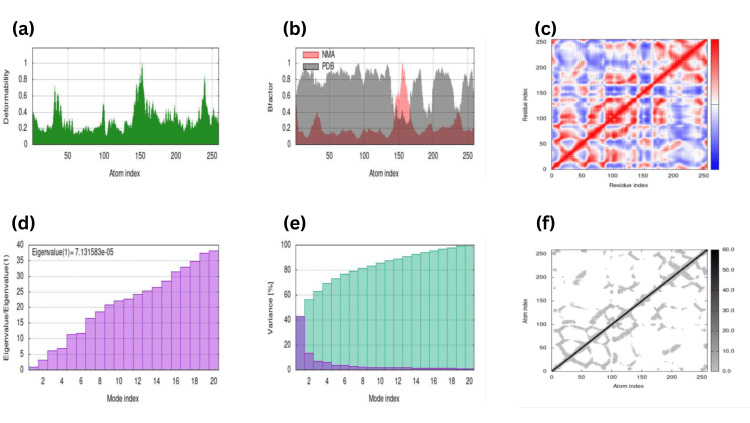
Molecular dynamic simulation done through iMODS of the Carnosol against PTPN11 receptor depicting (a) Deformability showing 4 hinges, maximum at 1, 1 hinges at 0.85, 1 hinges at 0.7 and least at 0.5 (b) Bfactor showing almost overlapping between predicted and experimental graph (c) Eigenvalue(1)=7.131e-05 (d) Variance (e) Covariance showing correlated regions around the central line in red (f) Elastic network in grey around the central line more flexibility meaning less deformability.

MD simulation analyses were carried out, resulting in the interactions study between the best-docked complex to check the elasticity and advancement in the receptor-ligand structures, as shown in (Figure 14-15).

The noticeable change in the variability and elasticity in the protein structures are depicted in the form of graphs on properties like Mobility deformability, Bfactor, Eigenvalue, etc., highlighting CASP8 and PTPN11 against RA eigenvalue 4.545e-05 (Figure 14c) and Carnosol with eigenvalue 7.131e-05 (Figure 15c).

## Discussion

In present times, AD is one of the major causes of dementia, risking life in people aged above 65 years. Our study observed a group of hub genes that qualified significant differential expressional behavior under neurodegenerative disorders. Of the selected hub genes, two important hub genes, CASP8 and PTPN11, play a crucial role in AD. Similar observations were found, providing supportive evidence that CASP8 activation is demarcated as one of the early events in apoptotic cascade and also contributed to extensive immunolabelling of tau accumulated neurons of AD cases [8]. CASP8 gene minimizes the accumulation of Aβ in the hippocampal region. Also, similar research shows the essential features of PTPN11 in the modulation of neurite in cultured neurons, resulting in increased programmed cell death [7]. The hub gene PTPN11 present in AD case is an upregulated gene; hence its over-expression leads to aggregation of Aβ in the hippocampal region of the brain, which is mainly responsible for the memory and learning process [24]. Thus, based on behavioral properties, CASP8 and PTPN11 were screened for target receptors. An extensive bibliographic study revealed that various natural bioactive compounds are being widely used as therapeutic drug candidates in treating various neurodegenerative disorders like AD. RA and Carnosol bio-active compounds were found to be effective ligands present in the Salvia Rosmarinus plant [25] with medicinal properties, which were screened based on ADMET properties and further docked against our target receptors. RA-CASP8 showed -8.0 kcal/mol followed by Donepezil (control) with -7.3 kcal/mol and Carnosol-PTPN11 having binding energy -9.0 kcal/mol w.r.t Tacrine (control) with value -8.0 kcal/mol. Similar work has been reported identifying the use of bio-active compounds Rutaecarpine against CASP8, showing an effective docking score of -6.13 kcal/mol [26]. Similarly, in the case of PTPN11, bioactive ligand palmitic acid showed a binding affinity of -7.3 kcal/mol [27], while for ligand dodecanoic acid, the score reflected was -7.09 kcal/mole [28].

Antioxidants are essential in various therapeutic strategies, including NRF2 pathway activation and regulation of anti-inflammatory genes. Research also suggests that one of our potential ligands, carnosol, crosses the blood-brain barrier and piles up in mammalian cells, which serves as a neuroprotective agent [25]. During neuronal differentiation in neurodegenerative diseases such as AD, PD, or FTD, tau proteins are modified and aggregated, thereby causing NFT to form and ultimately leading to neuronal death [29]. Our research reveals that the active antioxidant carnosol can be effectively docked with PTPN11, which can open new possibilities for exploring the mechanistic contribution of PTPN11 along with bioactive compounds in treating AD. MD simulation studies were also carried out for a docked complex of CASP8-RA and PTPN11-Carnosol, resulting in eigenvalue 4.545e-05 and 7.131e-05, respectively, predicting that CASP8-RA is more stable than PTPN11-Carnosol during MD simulation. Similarly, a study on E-protein with bio-active compounds Squash and Kunitz complex showed eigenvalue 8.980332e-06 and 2.013650e-04, making the E-Squash complex a stable compound [30]. The successful run of the docked complex along with MD provides effective possibilities for the use of natural-based antioxidant compounds as a potential drug candidate, which can be further validated by experimental research in treating neurodegenerative disorders such as AD.

Moreover, the limitation of our research through the computational approach highlighting ligand binding affinity prediction against potential target receptors has been theoretically qualified based on mathematical operations. However, there can be variation in actual results concerning theoretical results when tested in wet lab and clinical trial experimentation in multiple patients based on their demography, immunity, heredity, and epigenetic factors.

## Conclusions

AD is one of the most common causes of dementia in older people and causes a rapid decline in cognitive functions of the brain. Through our in-silico research, we identified clusters of potential hub genes, out of which CASP8 and PTPN11 showed vital participation in metabolic pathways targeting AD backed up with various research. These targeted genes were effectively docked as receptors against bio-active compounds RA and carnosol, having antioxidant and anti-inflammatory properties against standard drugs Donepezil and Tacrine as a control against AD with computational-based MD simulation validation. This computational analysis will open a wide range of possibilities for futuristic researchers in the identification of various undiscovered potent natural bio-active plant-based compounds that can show targeted behavior in curing this challenging neurological disorder as AD with multi-trial wet lab validations.

## Appendices

**Table 5:** Different bio active compounds showing logp, TPSA etc values in drulito.

S.No.	Title	MV	logP	AlogP	HBA	HBD	TPSA	AMR	Nrb	nAtom	nAcidicGroup	RC	nRigid	nAromRing	Nhb	Salerts
1	5281232	328.17	4.472	4.581	4	2	74.6	103.8	8	48	2	0	15	0	6	2
2	5281233	976.38	-1.214	-2.722	24	14	391.2	233.49	20	132	0	4	51	0	38	4
3	130796	330.17	-0.207	-0.692	7	4	116.45	81.41	4	49	0	2	20	0	11	1
4	61041	150.1	2.45	2.229	1	0	17.07	48.37	1	25	0	1	10	0	1	1
5	5281855	302.01	1.366	-1.304	8	4	133.52	74.69	0	28	0	4	25	2	12	3
6	370	170.02	0.964	-0.721	5	4	97.99	41.77	1	18	1	1	11	1	9	1
7	5280343	302.04	1.834	-1.244	7	5	127.45	83.44	1	32	0	3	23	2	12	2
8	11294368	470.27	2.786	1.1	6	2	96.36	125.76	2	72	0	6	37	0	8	3
9	71312551	782.41	2.087	-3.117	15	9	245.29	190.23	9	117	0	7	52	0	24	3
10	21599443	898.49	3.185	-2.898	17	9	255.91	217.03	9	137	0	9	62	0	26	2
11	9876264	928.5	2.942	-3.409	18	10	276.14	223	10	141	0	9	63	0	28	2
12	91827005	928.5	3.164	-2.909	18	10	276.14	223.33	10	141	0	9	63	0	28	2
13	15515703	472.36	7.103	1.821	4	2	58.92	132.05	1	82	0	6	38	0	6	1
14	5280934	278.22	7.538	-0.098	2	1	37.3	80.28	13	50	1	0	6	0	3	2
15	5280450	280.24	7.865	-0.948	2	1	37.3	75.37	14	52	1	0	5	0	3	2
16	3086007	444.4	9.837	3.301	2	2	40.46	132.81	4	84	0	4	31	0	4	1
17	65126	332.2	5.474	0.592	4	3	77.76	93.98	2	52	1	3	24	1	7	1
18	442009	330.18	5.108	0.628	4	2	66.76	91.58	1	50	0	4	26	1	6	3
19	5281792	360.08	1.578	0.099	8	5	144.52	97.84	7	42	1	2	20	2	13	4
20	9064	290.08	0.852	-0.936	6	5	110.38	81.07	1	35	0	3	22	2	11	1
21	107905	442.09	2.636	-1.049	10	7	177.14	119.35	4	50	0	4	31	3	17	3
22	72277	306.07	1.2	-1.499	7	6	130.61	82.67	1	36	0	3	23	2	13	1
23	65084	306.07	1.2	-1.499	7	6	130.61	82.67	1	36	0	3	23	2	13	1
24	5315472	308.1	1.887	1.519	4	2	74.6	98.41	6	39	0	2	18	2	6	2
25	969516	368.13	1.945	0.522	6	2	93.06	111.7	8	47	0	2	20	2	8	2
26	5469424	338.12	1.916	1.02	5	2	83.83	105.05	7	43	0	2	19	2	7	2

**Table 6:** Bio active compounds showing drug likeliness , drug score etc values in Osiris.

Title	ClogP	Solublity	Mol Weight	TPSA	Druglikeliness	Drug Score	Mutagenic	Tumorigenic	Irritant	Reproductive Effective
5281232	5.45	-2.61	328	74.6	1.24	0.56				
5281233	-1.9	-1.41	976	391.2	-1.85	0.28				
130796	-0.57	-1.52	330	116.4	-6.9	0.28			R	
61041	1.84	-1.99	150	17.07	-4.39	0.29			R	
5281855	1.28	-3.29	302	133.5	-1.6	0.51				
370	0.11	-0.74	170	97.99	0.12	0.27	R			R
5280343	1.49	-2.49	302	127.4	1.6	0.3	R	R		
11294368	2.56	-4.53	470	96	-0.63	0.21		O	O	O
71312551	-0.03	-4.15	782	245.2	-2.01	0.24				
21599443	0.95	-4.78	898	255.9	-6.65	0.12			R	
9876264	0.35	-4.65	928	276.1	-6.91	0.12			R	
91827005	0.4	-4.87	928	276.1	-6.56	0.12			R	
15515703	5.4	-5.64	472	58.92	0.37	0.18			R	
5280934	6.21	-4.09	278	37.3	-20.74	0.25				
5280450	6.47	-4.32	280	37.3	-25.56	0.24				
3086007	7.5	-6.3	444	40.49	-1.3	0.1			R	
65126	3.75	-4.24	332	77.76	-5.43	0.35				
442009	3.45	-4.17	330	66.76	-3.83	0.37				
5281792	1.45	-2.23	360	144.5	-2.07	0.49				
9064	1.51	-1.76	290	110.3	1.92	0.87				
107905	2.4	-2.46	442	177.1	2.81	0.75				
72277	1.16	-1.47	306	130.6	1.1	0.82				
65084	1.16	-1.47	306	130.6	1.1	0.82				
5315472	3.09	-3.59	308	74.6	-4.48	0.41				
969516	2.95	-3.62	368	93.06	-3.95	0.4				
5469424	3.02	-3.6	338	83.83	-3.55	0.41				
